# Retrospective Analysis of the Burden of Illness of Eosinophilic Granulomatosis With Polyangiitis (EGPA) Versus Asthma in Commercially Insured US Patients

**DOI:** 10.7759/cureus.42241

**Published:** 2023-07-21

**Authors:** Christopher F Bell, Mayank Ajmera, Juliana Meyers

**Affiliations:** 1 US Value Evidence and Outcomes, GSK, Durham, USA; 2 Health Economics, RTI Health Solutions, Durham, USA

**Keywords:** healthcare resource utilization, real world, egpa, cost, churg-strauss syndrome, asthma

## Abstract

Background and aim

Eosinophilic granulomatosis with polyangiitis (EGPA) is a rare inflammatory disorder associated with the presence of blood and tissue eosinophilia, extravascular granulomas, and asthma. Currently, the burden of EGPA on the patient and the healthcare system is not well characterized. This study aimed to assess the real-world clinical and economic burden of disease in adult patients with EGPA compared with matched patients with asthma without EGPA.

Methods

This retrospective cohort study used medical, pharmacy, enrolment, and demographic data from a US administrative claims database (PharMetrics Plus). Patients ≥18 years old, with ≥six months of continuous health plan enrolment in the baseline period, and ≥12 months of continuous health plan enrolment in the follow-up period were eligible for this analysis. Patients with EGPA and patients with asthma without EGPA were identified using diagnosis codes and were subsequently matched 1:5 (e.g., one patient with EGPA matched with five patients with asthma, without EGPA) based on baseline characteristics. The primary outcome measure was all-cause healthcare costs; secondary outcomes included healthcare resource utilization, medication usage, and clinical characteristics.

Results

In the final matched cohorts, there were 7183 patients with EGPA and 35,915 patients with asthma without EGPA. During the follow-up period, mean total all-cause healthcare costs were significantly higher in patients with EGPA than in those with asthma without EGPA (mean {standard deviation}: $44,405 {$82,060} vs $24,487 {$54,691}; p<0.0001). Patients with EGPA had mean total all-cause healthcare costs that were 73.9% greater than those in patients with asthma without EGPA, even after applying a multivariable analysis to adjust for differences in demographic and clinical characteristics. Medication usage was consistently higher in the EGPA population than in the asthma population (excepting short-acting β_2_-agonists). The majority of patients in the EGPA population (83.1%) also experienced ≥one relapse during the study period, with 26.3% of patients in the EGPA population experiencing a major relapse.

Conclusions

There is a significantly greater economic and clinical burden associated with EGPA compared with asthma without EGPA in adults. These results underscore the unmet need in this patient population for improved disease control strategies that will reduce the burden of EGPA on patients and the healthcare system.

## Introduction

Eosinophilic granulomatosis with polyangiitis (EGPA), formerly Churg-Strauss syndrome, is a systemic vasculitis, the key features of which include blood and tissue eosinophilia, the formation of extravascular granulomas, and asthma [1-3]. EGPA is often associated with sinusitis, pulmonary infiltrates, and peripheral neuropathy, and is characterized by periods of remission interspersed with symptomatic relapses [3-5]. EGPA remission has been defined as the absence of clinical systemic manifestations, with the exception of asthma, and ear, nose, and throat (ENT) symptoms, with minimal oral corticosteroid (OCS) and immunosuppressant use [6]. Relapses are characterized by the new appearance, recurrence, or worsening of clinical EGPA manifestations (excluding asthma and ENT symptoms) that require a change of therapy, or a dose increase [6]. The prevalence of EGPA is estimated to be between 2-38 cases per million people globally and between 3-31 per million people in the USA [7-12], and the incidence is estimated at between 0.18 and 4.0 cases per million person-years, depending on the geographic region and diagnostic criteria employed [8,11,13].

Treatment of EGPA is focused on reducing inflammation, suppressing the immune response, and reducing complications, with the aim of preventing relapses and increasing the time spent in remission [14]. The standard of care for EGPA involves treatment with corticosteroids alone or in combination with immunosuppressants, but the long-term use of these agents is associated with a significant side effect burden [6,14-16]. While corticosteroids, with or without immunosuppressant therapy, may help patients achieve and maintain remission, the relapsing, remitting disease course of EGPA commonly leads to patients relapsing following their initial remission [5,11]. Relapse can increase the risk of permanent tissue damage, organ injury, and impairment, which can lead to life-threatening complications [5,16,17]. Immunosuppressive agents, such as azathioprine, cyclophosphamide, or methotrexate, are often required as an add-on therapy to maintain or induce remission in organ- or life-threatening EGPA for patients with recurrent disease or for whom the corticosteroid dose cannot be tapered to less than 7.5 mg daily after three to four months of treatment [6,15].

In 2021, the American College of Rheumatology published guidelines for the management of EGPA; however, owing to the limited number of randomized controlled trials in EGPA, these recommendations rely on indirect evidence including expert opinion [18]. The lack of high-quality evidence-based guidelines and the relative scarcity of approved treatment options beyond corticosteroids and immunosuppressants further complicates the treatment of this rare disease [6,15,18,19].

To date, there is a paucity of published data on the real-world burden of illness associated with EGPA. This is due in part to the absence before October 2015 of EGPA-specific diagnosis codes, and the relative rarity of this condition. In addition, the criteria for EGPA diagnosis, relapses, and remission are subject to debate, making it challenging to accurately assess the burden of this illness, in terms of both patient quality of life and economic consequences [6]. The aim of this retrospective cohort study was to examine the clinical and economic burden associated with EGPA in a real-world setting, relative to patients with asthma without EGPA, using data from a US commercial insurance claims database.

Parts of this study have been previously presented as an abstract and as a poster at the American College of Rheumatology Convergence 2021 [20].

## Materials and methods

Study design

**Figure 1:**
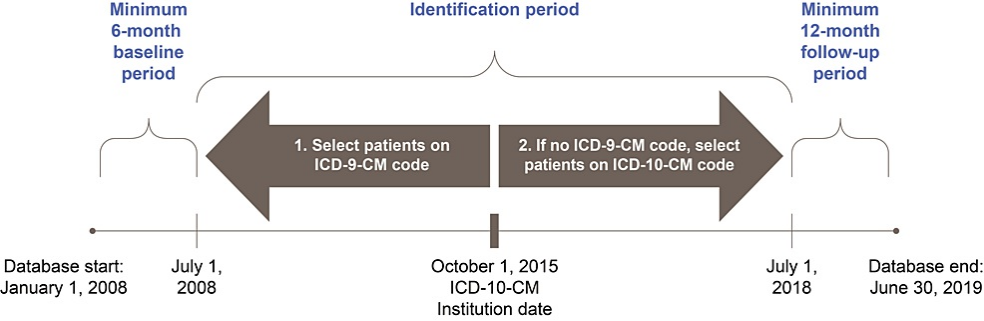
Study design of this retrospective cohort study conducted using administrative claims data. ICD-9-CM: International Classification of Diseases, Ninth Revision, Clinical Modification; ICD-10-CM: International Classification of Diseases, Tenth Revision, Clinical Modification

This was a retrospective cohort study (GSK study ID: 208438) conducted using administrative claims data from the IQVIA PharMetrics Plus database. This database contains data from more than 190 million patients in the United States since 2006 [21]. The database includes information on patient demographic characteristics, health plan enrolment, diagnoses, hospitalizations, laboratory testing, procedures, physician visits, prescriptions, and healthcare costs. Patients from every metropolitan statistical area of the United States are included in the database, with coverage from 90% of US hospitals and 80% of US doctors. Patients ≤18 years of age with EGPA and those with asthma without EGPA were identified in the PharMetrics Plus database between July 1, 2008, and July 1, 2018 (identification period) (Figure 1). The index date was the date of the first documented claim for vasculitis (prior to October 1, 2015) or EGPA (after October 1, 2015), within the identification period. The baseline period spanned six months prior to the index date and was used to assess baseline demographic, clinical characteristics, and healthcare resource utilization (HCRU). The follow-up period spanned 12 months after the index date and was used to assess the study outcomes.

Patients

Eligible patients were ≥18 years of age in the index year, with continuous enrolment with both medical and pharmacy coverage for ≥six months prior to the index date and ≥12 months following the index date. Patients with EGPA were identified using a combination of diagnosis, procedure, and medication codes, depending on the date of the initial identifying claim, owing to changes in the International Classification of Diseases (ICD) codes partway through the study period. There was no specific ICD, Ninth Revision, Clinical Modification (ICD-9-CM) code for EGPA, and a specific diagnosis code for EGPA was introduced in the tenth revision (ICD-10) on October 1, 2015. Therefore, from the start of the study period until October 1, 2015, the EGPA population was identified using seven previously published algorithms, which employed a combination of ICD-9-CM diagnosis codes, Current Procedural Terminology codes, and pharmacy claims for certain medications and specialties [22,23]. Criteria for each algorithm are shown in Table 1. Patients who met all the criteria of ≥1 algorithm in a 12-month period were included in the EGPA population. The index date was the date of the first qualifying claim for vasculitis within the identification period. For claims made after October 1, 2015, patients were identified if there was evidence of ≥1 medical claim with the ICD-10-CM diagnosis code for EGPA (M30.1 polyarteritis with lung involvement {Churg-Strauss}) [24]. Patients identified in the ICD-9-CM period were combined with those identified in the ICD-10-CM period to obtain the final EGPA population. There was no requirement for patients to be newly diagnosed during the identification period.

**Table 1:** Criteria for identification of patients with EGPA prior to October 1, 2015. *At the beginning of the identification period. ^†^For the purposes of this analysis, the author's specification of ≥3 asthma medication dispensings has been replaced with evidence of a medical claim with asthma ICD-9 diagnosis code (493.x) in any position. ^‡^Presence of ICD-9-CM diagnosis code for asthma or eosinophilia. ^§^Presence of ICD-9-CM diagnosis code for asthma and eosinophilia in any position (same or different claim). ^¶^At least two inpatient claims, at least three months apart, with the ICD-9-CM diagnosis code for vasculitis (446.4) in any position. **Rheumatologist, allergist/immunologist, nephrologist, pulmonologist, or otorhinolaryngologist. EGPA: eosinophilic granulomatosis with polyangiitis; ICD-9-CM: International Classification of Diseases, Ninth Revision, Clinical Modification; ICD-10-CM: International Classification of Diseases, Tenth Revision, Clinical Modification; OCS: oral corticosteroid; PPV: positive predicted value

Criterion	Author and algorithm number						
	Harrold #1	Harrold #2	Harrold #3	Harrold #4	Sreih #1	Sreih #2	Sreih #3
Age ≥18 years*	✔	✔	✔	✔	✔	✔	✔
Asthma diagnosis^†^	✔	✔	✔	✔	✔^‡^	✔^‡^	✔​​​​​​​^§^
Vasculitis	✔	✔	✔	✔​​​​​​​^¶^	✔	✔	✔
Mononeuritis	✔	-	-	-	-	-	-
Eosinophilia	-	✔	-	-	✔​​​​​​​^‡^	✔​​​​​​​^‡^	✔​​​​​​​^§^
Neurological symptoms	-	-	✔	-	-	-	-
Physician specialty**	-	-	-	-	✔	✔	-
Immunosuppressant prescription, including OCSs	-	-	-	-	-	✔	-
Reported PPV	100	80	40	10	27-90	27-90	100

The asthma without EGPA population consisted of patients who did not meet the criteria for EGPA but who had ≥1 medical claim with an ICD-9-CM (493.x) or ICD-10-CM (J45.xx, T486X5A, T486X5D, T486X5S, T486X6A, T486X6D, T486X6S) diagnosis code for asthma in a continuous 12-month period during the identification period. The index date was the date of the first qualifying asthma claim within the identification period.

Throughout this article, the overall unmatched patient groups are referred to as populations (all qualifying patients with EGPA and qualifying patients with asthma without EGPA) and the matched patient groups as cohorts. Patients with undefined sex were excluded from the matched cohorts (as sex was a matching criterion).

Endpoints and assessments

The primary objective of this analysis was to assess per-patient all-cause healthcare costs (costs associated with all claims, regardless of diagnosis) among patients with EGPA and patients with asthma without EGPA during the 12-month follow-up period. Healthcare costs were defined as the combined health plan-paid and patient-paid amounts; costs for inpatient visits, emergency department (ED) visits, outpatient hospital visits, office visits, and other outpatient visits, and pharmacy visits were reported, as well as the total healthcare costs. Healthcare costs were adjusted to 2019 US dollars using the annual medical care component of the Consumer Price Index.

Secondary objectives included the assessment of demographic and clinical characteristics (including medication utilization), all-cause HCRU, and healthcare costs among patients with EGPA and those with asthma without EGPA during the baseline period. Clinical (EGPA-related relapses) and economic (all-cause HCRU) outcomes in both cohorts during the follow-up period were also assessed. All-cause HCRU was defined as the number of inpatient visits, ED visits, outpatient hospital visits, office visits, other outpatient visits, and pharmacy visits. Baseline clinical characteristics assessed included the proportion of patients with each of the individual comorbidities from the Charlson Comorbidity Index (CCI); patient CCI scores calculated based on diagnosis codes used in medical claims during the baseline period; and the proportion of patients with ≥1 prescription for medications of interest, including asthma medications (biologics, inhaled anticholinergics, inhaled corticosteroids {ICSs} or ICSs plus long-acting β2-agonists {LABAs}, leukotriene modifiers, LABAs, long-acting muscarinic antagonists, mast cell stabilizers, methylxanthines, methylxanthine combinations, monoclonal antibodies, oral {systemic} corticosteroids {OCSs}, short-acting β2-agonists {SABAs}, and SABA and anticholinergic combinations) as well as biologic agents for autoimmune indications (in the case of patients with EGPA) [25-27]. Immunosuppressant use was included within the definition of biologics for both patient cohorts.

An algorithm developed by Raimundo et al. was used to identify EGPA-related major relapses and was also modified to include respiratory symptoms (e.g., asthma-related exacerbations) to identify EGPA-related relapses [28]. An EGPA-related relapse was defined as an event meeting any of the following seven criteria, occurring ≥30 days after the index date: (1) ≥one inpatient, ED or outpatient claim for vasculitis (prior to October 1, 2015) or EGPA (on or after October 1, 2015); (2) ≥one inpatient, ED, or outpatient claim for a relapse-associated condition based on major items in the Birmingham Vasculitis Activity Score; (3) ≥one inpatient claim with an asthma diagnosis in the primary position; (4) ≥one ED claim with an asthma diagnosis in the primary position that resulted in an inpatient stay ≤1 day; (5) ≥one ED claim (that did not result in an inpatient stay ≤one day) or outpatient claim with an asthma diagnosis in the primary position, and ≥one claim for an OCS ≤seven days of the claim date; (6) ≥one outpatient claim with allergic rhinitis, nasal polyposis, or acute/chronic sinusitis in the primary position that also includes either a new OCS claim, a new immunosuppressant claim, or an increase in OCS dosage to >4 mg/day ≤seven days of the claim date; or (7) ≥one inpatient claim with allergic rhinitis, nasal polyposis, or acute/chronic sinusitis in the primary position [29]. An EGPA-related major relapse was defined as an inpatient admission for any reason that occurred ≥30 days (and ≤one year) after the index date.

Sample size and statistical analysis

Each patient in the EGPA cohort was matched with five patients in the asthma cohort using direct covariate matching. The cohorts were matched based on age at the index date, year of the index date, sex, geographic region, and CCI score. Study variables including demographic characteristics (e.g., index month and year, age, age group, sex, geographic region), clinical characteristics (e.g., CCI score), medication utilization, relapses, and HCRU were analyzed descriptively (counts {N} and percentages for categorical variables, and mean and standard deviation {SD} for continuous variables).

Multivariate analysis was conducted to estimate adjusted total all-cause healthcare costs, EGPA-related healthcare costs, and asthma-related healthcare costs for the EGPA and asthma cohorts. Adjusted healthcare costs were estimated using a generalized linear model with a gamma distribution and log link. This analysis included total healthcare costs in the follow-up period as the dependent variable, and healthcare cost quartiles in the baseline period, baseline demographics, and clinical characteristics as independent variables. Adjusted healthcare costs were presented using both the least-squares means and predicted values approach.

Patient and public involvement and ethics approval

No direct patient contact or primary collection of individual human subject data occurred for this analysis, and patients were de-identified in the PharMetrics Plus database. As the data source required no direct patient contact or primary collection of individual human patient data and was anonymized, informed consent and ethics committee or institutional review board approval were not required.

## Results

Patient populations

Overall 11,280 patients were identified as having EGPA based on ≥one of the ICD-9-CM algorithms and 878 patients were identified as having EGPA based on the ICD-10-CM diagnosis codes. Of these, 7197 met the eligibility criteria and were included in the unmatched EGPA population. A total of 3,490,566 patients were identified as having asthma without EGPA, met the eligibility criteria, and were included in the asthma population. After applying 1:5 direct covariate matching, a total of 7183 patients and 35915 patients were included in the matched EGPA and asthma without EGPA cohorts, respectively.

Demographics, clinical characteristics, all-cause HCRU, and associated costs during the six-month baseline period

In the matched cohorts, mean age was approximately 51 years at the index date, just under three-quarters of patients were female, and the mean CCI score during the baseline period was 2.8 (Table 2). The most common comorbidity in the baseline period for patients in the EGPA cohort was chronic pulmonary disease (56.8%), and for patients in asthma without EGPA cohort this was hypertension (53.0%) (Table 2). The proportion of patients receiving medications of interest in the baseline period was greater in the EGPA cohort than in the asthma without EGPA population, with more than twice as many patients in the EGPA population receiving an OCS, when compared with the asthma without EGPA population (Table 2). The three most common medications used in the baseline period in the EGPA cohort were OCS (42.7%), ICS (31.8%), and SABA (31.4%), compared with SABA (25.1%), ICS (19.9%), and OCS (19.0%) in asthma without EGPA cohort (Table 2). The proportion of patients utilizing each care setting (inpatient visits, ED visits, outpatient hospital visits, office visits, other outpatient visits, and pharmacy visits) in the baseline period and associated costs in each care setting were consistently higher in the EGPA cohort than in asthma without EGPA cohort (Table 2). Mean (SD) total healthcare costs in the baseline period were higher in the EGPA cohort than in asthma without EGPA cohort ($19,610 {$46,007} vs $11,521 {$33,138}) (Table 2).

**Table 2:** Demographics and clinical characteristics; all-cause HCRU and associated costs; and medication usage during the six-month baseline period for the EGPA and asthma without EGPA populations and matched cohorts. ^*^All-cause HCRU and costs include all medical visits, regardless of the underlying diagnosis or medication. Costs were adjusted to 2019 US dollars based on the medical care component of the Consumer Price Index. ^†^Patients with ≥1 prescription. ^‡^“Other” includes LABAs, long-acting muscarinic antagonists, methylxanthines, mast cell stabilizers, inhaled anticholinergics, SABA/anticholinergic combinations, methylxanthine combinations, monoclonal antibodies, biologics. ^§^Number of visits and costs measured across all patients, regardless of whether they had utilization in the care setting. ^¶^Other outpatient visit includes visits in other care settings (e.g., home healthcare), and laboratory services, among others. CCI: Charlson Comorbidity Index; ED: emergency department; EGPA: eosinophilic granulomatosis with polyangiitis; HCRU: healthcare resource utilization; ICS: inhaled corticosteroid; LABA: long-acting β_2_-agonist; OCS: oral corticosteroid; SABA: short-acting β_2_-agonist; SD: standard deviation

Characteristics	Overall populations (unmatched)		Matched cohorts	
	EGPA population N=7197	Asthma without EGPA population N=3,490,566	EGPA cohort N=7183	Asthma without EGPA cohort N=35,915
Age at index date, mean (SD)	51.0 (14.0)	44.5 (14.9)	51.0 (13.9)	51.2 (14.0)
Female, n (%)	5303 (73.7)	2,206,057 (63.2)	5294 (73.7)	26,470 (73.7)
Geographic region, n (%)				
Northeast	2132 (29.6)	943,753 (27.0)	2131 (29.7)	10,655 (29.7)
South	2158 (30.0)	1,077,126 (30.9)	2154 (30.0)	10,770 (30.0)
Midwest	1916 (26.6)	953,976 (27.3)	1914 (26.7)	9570 (26.7)
West	834 (11.6)	435,163 (12.5)	832 (11.6)	4160 (11.6)
Missing	157 (2.2)	80,548 (2.3)	152 (2.1)	760 (2.1)
CCI score, mean (SD)	2.8 (2.7)	0.9 (1.6)	2.8 (2.7)	2.8 (2.7)
Comorbidities, n (%)				
Chronic pulmonary disease	4089 (56.8)	411,089 (11.8)	4078 (56.8)	10,016 (27.9)
Connective tissue disease	1084 (15.1)	55,778 (1.6)	1078 (15.0)	1899 (5.3)
Hypertension	2897 (40.3)	725,187 (20.8)	2887 (40.2)	19,045 (53.0)
Depression	920 (12.8)	270,520 (7.8)	914 (12.7)	7499 (20.9)
Diabetes without complications	1222 (17.0)	304,767 (8.7)	1218 (17.0)	10,373 (28.9)
Skin ulcers/cellulitis	933 (13.0)	104,376 (3.0)	930 (13.0)	3387 (9.4)
All-cause HCRU*, n (%)				
≥1 inpatient visit	1571 (21.8)	157,544 (4.5)	1561 (21.7)	5053 (14.1)
≥1 ED visit	2333 (32.4)	498,975 (14.3)	2323 (32.3)	8915 (24.8)
≥1 outpatient hospital visit	5283 (73.4)	1,493,978 (42.8)	5270 (73.4)	22,343 (62.2)
≥1 office visit	6867 (95.4)	2,768,154 (79.3)	6854 (95.4)	33,085 (92.1)
≥1 other outpatient visit^§^	4414 (61.3)	1,315,984 (37.7)	4406 (61.3)	19,965 (55.6)
≥1 pharmacy visit	6105 (84.8)	2,638,807 (75.6)	6091 (84.8)	29,710 (82.7)
Medications, n (%)^†^				
SABA	2263 (31.4)	751,384 (21.5)	2254 (31.4)	9002 (25.1)
ICS	2288 (31.8)	584,393 (16.7)	2285 (31.8)	7157 (19.9)
ICS/LABA	1350 (18.8)	303,997 (8.7)	1347 (18.8)	3825 (10.7)
Leukotriene modifiers	1155 (16.1)	273,603 (7.8)	1153 (16.1)	2981 (8.3)
OCS	3078 (42.8)	484,842 (13.9)	3065 (42.7)	6811 (19.0)
Other^‡^	1079 (15.0)	171,206 (4.9)	1076 (15.0)	3542 (9.9)
All-cause healthcare costs, $, mean (SD)*				
Inpatient visit costs^§^	8818 (37664)	1055 (10,969)	8786 (37,661)	4570 (27,424)
ED visit costs^§^	580 (2018)	185 (1039)	578 (2017)	399 (2416)
Outpatient hospital visit costs^§^	4042 (13,784)	1002 (5378)	4033 (13,782)	2377 (9547)
Office visit costs^§^	2385 (6478)	857 (2363)	2387 (6484)	1485 (4179)
Other outpatient visits costs^§¶^	1353 (9279)	349 (3599)	1350 (9282)	896 (7136)
Pharmacy visit costs^§^	2477 (5528)	979 (3314)	2477 (5532)	1794 (4225)
Total healthcare costs^§^	19,655 (46,028)	4427 (14,909)	19,610 (46,007)	11,521 (33,138)

All-cause HCRU and associated costs per-patient during the 12-month follow-up period

**Figure 2:**
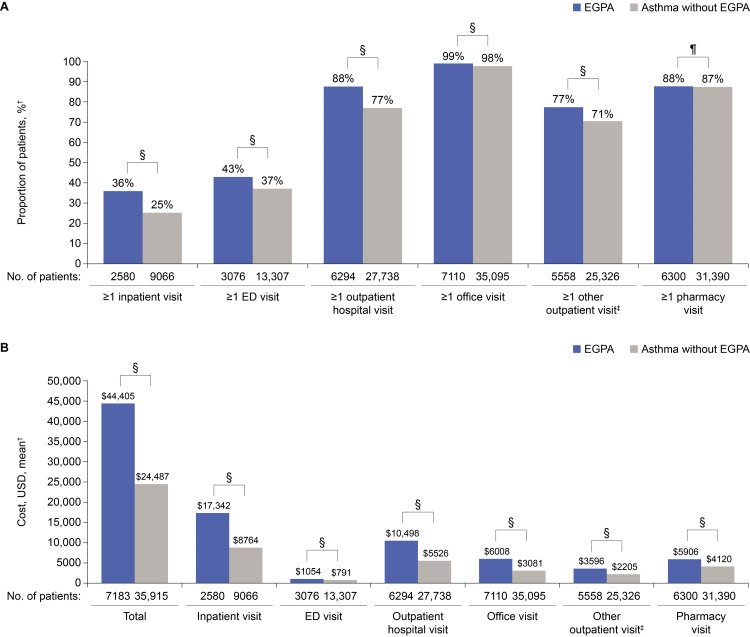
All-cause HCRU (A) and unadjusted all-cause costs (B) for the EGPA and asthma without EGPA cohorts during the 12-month follow-up period (matched cohorts).* *All-cause HCRU and costs include all medical visits, regardless of the underlying diagnosis or medication. Costs were adjusted to 2019 US dollars based on the medical care component of the Consumer Price Index. ^†^Number of visits and costs measured across all patients, regardless of whether they had utilization in the care setting. ^‡^Other outpatient visit includes visits in other care settings (e.g., home healthcare), and laboratory services, among others. ^§^P-value<0.0001. ^¶^P-value=0.4744. ED: emergency department; EGPA: eosinophilic granulomatosis with polyangiitis; HCRU: healthcare resource utilization; USD: US dollars

HCRU was higher in the EGPA cohort than in asthma without EGPA cohort during the 12-month follow-up period (Figure 2, panel A). A significantly greater (p<0.0001) proportion of patients with EGPA required ≥one all-cause inpatient visit, ED visit, outpatient hospital visit, office visit, and other all-cause outpatient care over the 12-month follow-up period. No significant difference was observed between the number of patients with ≥one pharmacy claim in the EGPA and asthma without EGPA cohorts; however, there was a significantly greater (p<0.0001) number of pharmacy claims on average in the EGPA cohort than in asthma without EGPA cohort (mean {SD}: 47.4 {44.2} vs 38.8 {40.9}).

In the 12-month follow-up period, total unadjusted mean all-cause healthcare costs in the matched cohort were significantly higher for patients with EGPA than for those with asthma without EGPA (mean {SD}: $44,405 {$82,060} vs $24,487 {$54,691}; p<0.0001; Figure 2, panel B). In the EGPA cohort, the largest cost components were inpatient visits, outpatient hospital visits, followed by office visits, compared with inpatient visits, outpatient hospital visits, followed by pharmacy claims in asthma without EGPA cohort. After adjusting for patient demographic and clinical characteristics with a multivariable analysis, patients with EGPA had total all-cause healthcare costs in the follow-up period that were 73.9% greater than those observed with asthma without EGPA (rate ratio 1.74 {95% confidence interval (CI)}: 1.68, 1.79). Total adjusted mean all-cause healthcare costs in the follow-up period were $34,004 (95% CI: $33,097, $34,935) in the EGPA cohort and $19,552 (95% CI: $19,318, $19,788) in asthma without EGPA cohort.

Clinical outcomes during the 12-month follow-up period: medication and EGPA relapses

**Figure 3:**
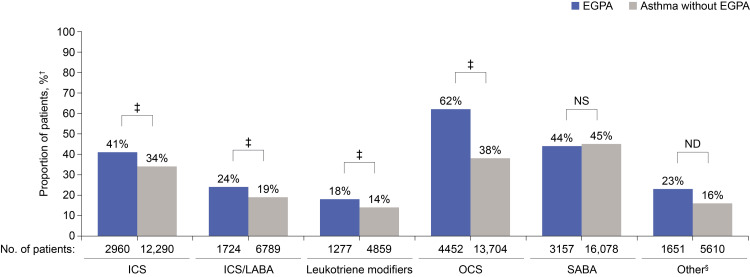
Medications received during the 12-month follow-up period (matched cohorts).* *The five most commonly used medications are shown individually, all other medications are grouped as “other.” ^†^Patients with ≥1 prescription. ^‡^p<0.0001. ^§^“Other” includes LABAs, long-acting muscarinic antagonists, methylxanthines, mast cell stabilizers, inhaled anticholinergics, SABA/anticholinergic combinations, methylxanthine combinations, monoclonal antibodies, and biologics. EGPA: eosinophilic granulomatosis with polyangiitis; ICS: inhaled corticosteroid; LABA: long-acting β_2_-agonist; ND: not determined; NS: not significant; OCS: oral corticosteroid; SABA: short-acting β_2_-agonist

In the follow-up period, medication use was generally significantly higher in the EGPA cohort than in asthma without EGPA cohort, except that a numerically slightly greater proportion of patients with asthma without EGPA received SABA than those with EGPA (44.8% vs 44.0%; p=0.24) (Figure 3). The three most frequently used medications in the EGPA cohort were OCS (62.0%), SABA (44.0%), followed by ICS (41.2%) compared with SABA (44.8%), OCS (38.2%), followed by ICS (34.2%) in asthma without EGPA cohort (Figure 3).

The majority of patients (83.1%) in the EGPA cohort had an EGPA-related relapse in the 12-month follow-up period, with a mean (SD) of 10.5 (20.5) observed relapses. On average, the first relapse was observed approximately three months after the index date. In the EGPA cohort, 26.3% of patients had ≥one EGPA-related major relapse, with a mean (SD) of 2.2 (2.4) major relapses among patients with ≥one major relapse during the 12-month follow-up. On average, the first major relapse occurred approximately five months after the index date. The three most common primary or non-primary diagnosis codes recorded during inpatient admissions associated with EGPA-related major relapses were hypertension (20.9%), asthma (14.5%), and pneumonia (10.6%).

## Discussion

Comprehensive data on the burden of disease in EGPA are currently lacking, owing to the rarity of the disease, the scarcity of approved treatments, and the absence of specific diagnosis codes. To address this knowledge gap, we compared data from patients with EGPA with a matched cohort of patients with asthma without EGPA to quantify the clinical and economic burden of EGPA. An asthma control group was selected for this analysis as asthma is a key clinical symptom of EGPA [3,6], and because the disease burden in asthma is already well understood [30-32]. As such, asthma without EGPA population represents a useful point of comparison to evaluate the incremental impact of EGPA on patients and payers. However, it should be noted that the matched asthma cohort differed at baseline in several ways from the unmatched asthma population, in terms of sex, CCI score, comorbidities, HCRU, medication usage, and healthcare costs. This may be due to the fact that the EGPA and asthma without EGPA cohorts were matched based on CCI score, which likely led to the selection of a subsection of patients with more severe disease than the unmatched asthma population.

During follow-up, the unadjusted all-cause mean healthcare costs associated with EGPA were almost twice as high as those associated with asthma without EGPA ($44,405 vs $24,487) and were comparable to the per-patient costs associated with EGPA or GPA in other real-world studies [4,28]. Even after adjusting for demographic and clinical factors, total all-cause mean healthcare costs in the EGPA cohort were almost double compared to those in asthma without EGPA cohort ($34,004 vs $19,552). These costs are considerably greater than the mean all-cause healthcare costs associated with diseases, such as type 2 diabetes, rheumatoid arthritis, and knee osteoarthritis [33-35]. Furthermore, patients with EGPA utilized a significantly greater number and proportion of healthcare services in all settings, including hospital inpatient, outpatient and ED visits, and physician office visits than patients with asthma without EGPA. Medication usage was also higher in patients with EGPA, with a more than 60% increase in the number of patients receiving ≥one prescription for an OCS in the EGPA population compared with asthma without EGPA population. The increased HCRU and medication use observed in this analysis in patients with EGPA compared with patients with asthma without EGPA may reflect the inherently increased disease severity associated with EGPA.

Our overall findings are in agreement with recent studies in this area. In a previous analysis using a different administrative claims database, mean annual all-cause healthcare costs for patients with EGPA of $49,593 were reported, which is comparable to the total all-cause costs reported here [4]. Similarly, one previous analysis examining healthcare costs in GPA, a related condition, recorded annual all-cause healthcare costs of $41,400 [28]. A separate analysis also established a similar level of medication usage (74.5-78.4% of patients with EGPA had ≥one OCS prescription, compared with 62.0% of patients in this analysis) and HCRU (30.7-30.8% of patients experienced a hospitalization and 43.1-45.2% experienced an ED visit, compared with 35.9% and 42.8%, respectively, in our analysis) [7].

Periods of remission interrupted by intermittent relapses are important characteristics of EGPA, and relapses are of clinical importance because they are associated with an increased risk of permanent organ damage [16]. However, the precise parameters of EGPA relapse and remission are subject to debate, and there are no specific codes for identifying relapses [6,24]. Using a modified version of a previously reported algorithm for identifying GPA-related major relapses, we found that 83.1% of patients with EGPA experienced ≥one relapse during the follow-up period, with an average of 11 relapses during this time [28]. In addition, 26.3% of patients experienced a major EGPA relapse (mean per annum: 2.2). Hypertension was one of the main diagnosis codes observed during inpatient admissions linked with major EGPA relapse. Cumulative doses of oral glucocorticoids have been associated with an increased incidence of hypertension in adults with chronic inflammatory diseases [36]. It is therefore possible that the hypertension observed here may be a side effect of OCS use and not an indicator of EGPA relapse. The frequency of relapse observed in the current analysis is slightly different than that has been previously reported; a previous analysis found that 44.1% of patients experienced ≥one relapse, and 35.2% experienced ≥one major relapse in a 12-month period [4]. Given that a similar methodology was used in these two analyses, the difference observed may be due to the use of different databases and study populations. Although both databases included information from patients with commercial health insurance plans, there were some notable differences between the baseline patient characteristics; patients in the current analysis had higher mean CCI scores, younger average age, and were slightly more likely to be female. By comparison, in the clinical trial setting, relapse rates of 82% were observed among patients with EGPA receiving placebo over a 12-month period [37]. While not measured in the current analysis, a previous cost analysis estimated the annual cost of EGPA among patients with and without evidence of relapse and found that there was an approximately three-fold increase in total all-cause healthcare costs in patients who experienced a relapse compared with patients who did not [4]. In the case of major relapses, there was an approximately four-fold increase in total costs in these patients compared with patients who did not experience relapse [4]. Similarly, another analysis found that patients with GPA who experienced a relapse had mean all-cause healthcare costs of $88,631 over the 12-month follow-up, while those that did not experience a relapse incurred costs of $32,005 [38]. Taken together, these results highlight the need for effective therapies to reduce the frequency of relapses in patients with EGPA, which in turn would likely result in decreased HCRU and associated costs.

More recently, novel therapies have demonstrated efficacy in this regard. For example, mepolizumab, a humanized, anti-interleukin-5 monoclonal antibody, has been approved for the treatment of patients with severe asthma, hypereosinophilic syndrome, EGPA, and chronic rhinosinusitis with nasal polyps [39]. In patients with EGPA, this agent has been shown to significantly increase time spent in remission, as well as increase the proportion of patients who achieve remission and the time to first relapse when compared with standard of care [37,40]. Moreover, other products for the treatment of EGPA are currently being investigated in clinical development programs [41,42].

This analysis has several strengths, such as it included a quantitative assessment of relapses in patients with EGPA and included multivariable modeling to understand the incremental burden of EGPA on the healthcare system in comparison to asthma without EGPA. However, the analysis was limited by the absence of a specific ICD-9-CM code for EGPA [43]. As a solution, we used multiple published claims-based algorithms for the identification of patients with EGPA in the database prior to October 2015 [22,23]. However, this approach may have been overly broad, potentially leading to the inclusion of some patients in the EGPA population that did not have EGPA. Although the EGPA population may have included some patients without EGPA, all algorithms required an asthma diagnosis and evidence of vasculitis, as well as ≥one criterion that is consistent with EGPA (e.g., mononeuritis multiplex, eosinophilia, neurological symptoms, physician specialty, or immunosuppressant prescription, including OCSs) and so the number of miscoded patients is likely to be very low. As the population selected for analysis included newly diagnosed patients with EGPA with mild disease severity, the results presented here may not be representative of patients with relapsing EGPA in which immunosuppressant utilization is high and the use of rescue therapies, such as plasmatic exchanges, are more common. Furthermore, the use of administrative data itself has some inherent limitations [44]. Such data is intended for billing purposes, rather than for clinical or research purposes, and as such, access to laboratory data, in particular, is limited in administrative claims databases. The presence of a documented prescription in the database does not necessarily mean that the medication was taken as prescribed or taken at all. Diagnostic codes may be incorrectly or accidentally applied, or included as a rule-out criterion rather than as a record of an actual diagnosis. It is also of note that patients with EGPA often have frequent ENT involvement; however, a further limitation of this analysis is that ENT involvement was not specifically captured within the EGPA diagnostic criteria [45]. Furthermore, the database used contains only patients with commercial insurance and does not include uninsured patients, or patients enrolled in certain state-sponsored insurance programs (e.g., Medicaid). Therefore, the results may not be accurately reflective of the overall US population.

## Conclusions

Despite the limitations outlined in the above section, the analysis presented here demonstrates that EGPA is associated with a considerable burden of disease in terms of HCRU, costs, medication use, and relapses. Treatment of patients with EGPA incurs significantly greater HCRU and associated costs than the treatment of patients with asthma without EGPA. These findings underscore the need for improved disease control strategies to prevent relapses, maintain remission, and reduce the burden on the healthcare system, and may have clinical and policy implications for the treatment of patients with EGPA. Further research is required to refine clinical management strategies to address the unmet need in this patient population, and thereby reduce the significant burden on the healthcare system by reducing relapses and increasing time spent in remission.
